# Population-based study on hospital admissions for pediatric status asthmaticus: from before to after the COVID-19 pandemic

**DOI:** 10.3389/fped.2025.1534770

**Published:** 2025-05-09

**Authors:** Ka Ka Siu, Michael Kwan Leung Yu, Jaime S. Rosa Duque, Sophelia Hoi Shan Chan, Yu Lung Lau, So Lun Lee

**Affiliations:** ^1^ Department of Paediatrics & Adolescent Medicine, School of Clinical Medicine, The University of Hong Kong, Hong Kong, Hong Kong SAR, China; ^2^ Department of Paediatrics & Adolescent Medicine, Queen Mary Hospital, Hong Kong, Hong Kong SAR, China

**Keywords:** status asthmaticus, COVID-19, hospitalization, pediatric intensive care units, asthma

## Abstract

**Objectives:**

The increase in respiratory infections post-COVID-19 pandemic, attributed to relaxed masking and social distancing, has raised concerns about a new pattern of severe asthma exacerbations in children. We compare admission rates, severity, and risk factors of status asthmaticus in children with reference to the past 3 years before, during, and after the COVID-19 pandemic.

**Study design:**

This is a population-based cross-sectional analysis. Admission records were retrieved from the Clinical Data Analysis and Reporting System of the Hospital Authority in Hong Kong. Patients aged 2 to <18 years admitted for status asthmaticus between January 2017 and March 2024 were included.

**Main results:**

The incidence rate of pediatric status asthmaticus increased after the COVID-19 period compared to before COVID-19 (5.7–7.3 per 100,000 children aged 2 to <18 years), with a higher increase in children aged 2 to <6 years (10.1–20.6 per 100,000 children aged 2 to <18 years). There was a higher percentage of status asthmaticus admissions among total pediatric asthma admissions after COVID-19 (0.83% vs. 2.87%, *p* < 0.0001). Admissions are predicted to return to before COVID-19 levels by 2025.

**Conclusions:**

Status asthmaticus increased after the COVID-19 pandemic, particularly in preschoolers. Public health measures during the pandemic may have prevented the children's immune systems from being educated with infection.

## Introduction

Pediatric asthma is a prevalent health issue worldwide, and there is a growing trend in its incidence. The World Health Organization has estimated that there are presently around 300 million individuals with asthma globally, and this is expected to rise to 400 million by 2025 (1). According to CDC surveillance data, 40% of children with asthma in the United States have uncontrolled asthma (2). Episodes of progressive worsening of symptoms, termed exacerbations, may require an emergency department visit or hospital admission. Severe acute asthma or status asthmaticus refers to an asthma exacerbation that is refractory to conventional therapy and necessitates PICU admission for intravenous therapy, and potentially intubation and invasive ventilation (3). Childhood asthma was responsible for 12.9 thousand deaths worldwide in 2019 (4), contributing significantly to the global burden of disease.

Asthma exacerbation is linked to seasonal changes (5) and air pollution (6). Respiratory tract infections are also a common cause of asthma exacerbations in children (7). Coronavirus disease (COVID-19) caused by severe acute respiratory syndrome coronavirus 2 (SARS-CoV-2) was first reported in late 2019 and resulted in a pandemic (8). A children's hospital in the United States observed a 76% decrease in asthma-related emergency department utilization compared to pre-COVID, which was also well below historical seasonal variation (9). Another study from Hong Kong demonstrated that pediatric asthma hospitalization and respiratory virus isolation decreased during the first year of the COVID-19 pandemic due to non-pharmaceutical interventions such as mandatory mask-wearing and social distancing (10). Although the incidence of pediatric asthma hospitalization during the COVID-19 pandemic has been investigated, the incidence of status asthmaticus during and after the COVID-19 pandemic is not widely reported.

The primary outcome of this study was to analyze the admission rates of children with status asthmaticus to the pediatric intensive care units (PICUs) before, during, and after the COVID-19 pandemic in Hong Kong. The secondary outcomes included assessing the severity of status asthmaticus based on factors such as hospital length of stay, use of oxygen and ventilator support, intravenous magnesium sulfate (MgSO4) administration, pneumonia and mortality rate. The study also aimed to identify the risk factors for pediatric status asthmaticus admission, examine the age distribution of patients, and investigate the prevalence of respiratory virus infections in the patients. Hong Kong's strict public health measures implemented in response to COVID-19 provided a unique opportunity to observe how respiratory viruses affect children with status asthmaticus and the development of immune debt in this population.

## Materials and methods

### Study design

This study was a population-based cross-sectional analysis comparing the rate and severity of pediatric status asthmaticus hospital admissions before, during, and after the COVID-19 period in Hong Kong. The COVID-19 period was defined as January 23, 2020, to January 22, 2023 (36-months), starting from the first imported case from Wuhan to Hong Kong and covering the major epidemic waves (11).

The after COVID-19 period was defined as January 23, 2023, to January 22, 2024 (12-months), when COVID-19 was no longer recognized as a public health emergency of international concern by the World Health Organization (12). Data from January 23, 2017 to January 22, 2020 (36-months) were collected for comparison as before the COVID-19 period.

### Data source

The episode-based admission records were extracted from the Clinical Data Analysis and Reporting System (CDARS) of the Hospital Authority. The Hospital Authority is a public organization that provides 80% of inpatient services in Hong Kong (13). Among these hospitals, nine PICUs care for over 1.2 million children under age 18 and a population of 7.5 million in Hong Kong. Public hospitals under the Hospital Authority provide care to patients with COVID-19 and other acute admissions through emergency departments.

### Data collection

Data from January 2017 to March 2024 was collected using the International Classification of Diseases, Ninth Revision (ICD-9) coding (14). The study included 2 to <18 years children with following diagnoses: 1) status asthmaticus (ICD-9 codes: 493.01, 493.11 and 493.91) or 2) asthma (ICD-9 codes: 493) with PICU admission, or 3) wheezing [ICD-9 codes: 786.09 (5) and 786.09 (7)] with PICU admission. Exclusion criteria were patients with coexisting bronchiectasis and/or cystic fibrosis. Additionally, children aged <2 years were also excluded as these children were likely to have bronchiolitis, and differentiation between bronchiolitis and asthma is difficult (3). The number of monthly total pediatric asthma and total pediatric hospital admissions for the same age group were retrieved as control data. To prevent any false-negative cases of pediatric asthma, we included wheezing cases with PICU admission in the total pediatric asthma admission count.

Demographic data, clinical features and laboratory test results on nasopharyngeal specimens were collected for the analysis.

Monthly mean ambient temperature, rainfall and humidity were retrieved from the Hong Kong Observatory (15). Air quality data were collected from the Hong Kong Environmental Protection Department, represented by the monthly Air Quality Health Index (AQHI) (16). It was calculated based on the cumulative health risk attributable to 3-hour moving average concentrations of four air pollutants (namely, ozone [O_3_], nitrogen dioxide [NO_2_], sulfur dioxide [SO_2_], and particulate matter [PM_2.5_/PM_10_]). The AQHI was reported hourly in 13 stations located in different areas of Hong Kong. The time of high to serious grade AQHI was summed and expressed as a percentage of total hours in each month.

### Statistical analyses

The Kruskal–Wallis test was used to compare the median age and length of stay for status asthmaticus admissions between the three periods. Fisher's exact test was used to compare percentages of female, status asthmaticus admissions among total pediatric asthma and hospital admissions, clinical severity, viral distribution, and different age groups. Poisson regression was used to calculate the relative risk of status asthmaticus hospital admission changes during and after the COVID-19 periods, with the period before COVID-19 set as the baseline. The log-transformed monthly total pediatric admission was used as an offset criterion, while AQHI (% of high to serious), humidity (%), temperature (°C), and rainfall (mm) were set as covariates. Pearson's correlation was used to predict the trend of pediatric status asthmaticus admissions from April 2024 to March 2025, based on admissions data from February 2023 to March 2024. A *p*-value <0.05 was considered statistically significant. Data analyses were performed using IBM SPSS Statistics (version 27.0.1.0). Graphs were created using GraphPad Prism (version 10.2.2). Curve smoothing was performed by averaging the 10 neighbouring values using a 2nd order smoothing polynomial.

### Standard protocol approval

This study was approved by the HKU/Hospital Authority HK West Cluster Institutional Review Board Committee (reference number: UW24-254). This retrospective and anonymous study did not require informed consent from participants.

## Results

### Primary outcome

There were 52 pediatric status asthmaticus hospital admissions cases before the COVID-19 period (36-months), 46 cases during the COVID-19 period (36-months), and 65 cases after COVID-19 (12-months). No cases with coexisting bronchiectasis and/or cystic fibrosis were identified in this cohort. There was no significant difference in gender distribution and median age of patients across the three periods (Table 1).

**Table 1:** Comparison of demographic and clinical features of hospitalized children with status asthmaticus between before, during and after COVID-19 pandemic.

	COVID-19 period (number of status asthmatics cases)			*p*-value	
	Before (*n* = 52)	During (*n* = 46)	After (*n* = 65)	Before vs. after	During vs. after
Demographics					
Median age in years (range)	6.9 (2.1–17.7)	4.8 (2.0–17.8)	4.9 (2.2–17.6)	0.117	0.482
Female	29 (55.8)	19 (41.3)	32 (49.2)	0.577	0.444
Admission rates					
Monthly average of status asthmatics cases	1.45	1.28	5.44	–	–
Percentage among total paediatric asthma admissions	0.83	2.43	2.87	**<0**.**0001**	0.387
Percentage among total paediatric hospital admissions	0.04	0.06	0.11	**<0**.**0001**	**0**.**008**
Clinical severity					
Median LOS in days (range)	5.0 (2.0–26.0)	5.5 (1.0–178.0)	6.0 (0.0–47.0)	0.986	0.525
Use of MgSO_4_	20 (38.5)	18 (39.1)	28 (43.1)	1.000	0.700
Use of oxygen enrichment	19 (36.5)	29 (63.0)	55 (84.6)	**0**.**015**	**0**.**013**
Use of NIV	3 (5.8)	2 (4.3)	10 (15.4)	1.000	0.118
Use of IV	6 (11.5)	6 (13.0)	10 (15.4)	1.000	0.790
Pneumonia	14 (26.9)	9 (19.6)	21 (32.3)	0.477	0.193
Death	1 (1.9)	1 (2.2)	1 (1.5)	1.000	1.000

Data are presented as number (percentage) if unspecified.

The before COVID-19 period was defined as January 23, 2017, to January 22, 2020 (36 months). The during COVID-19 period was defined as January 23, 2020, to January 22, 2023 (36 months). The after COVID-19 period was defined as January 23, 2023, to January 22, 2024 (12 months). The total paediatric asthma admissions were 6,271 cases before COVID-19, 1,896 cases during COVID-19, and 2,268 cases after the COVID-19 period. The total paediatric hospital admissions were 139,851 cases before COVID-19, 71,997 cases during COVID-19, and 61,030 cases after the COVID-19 period. LOS, length of stay; IV, invasive ventilation; NIV, non-invasive ventilation.

Bold values indicate *p* < 0.05.

In comparison to the periods before and during COVID-19, we observed an increase in the incidence rate of children admitted for status asthmaticus after COVID-19 (Before: 5.7 vs. During: 5.2 vs. After: 7.3 per 100,000 children aged 2 to <18 years per year) (Table 2; Supplementary Figure 1). Particularly notable was the higher increase among children aged 2 to <6, with an incidence rate of 20.6 per 100,000 children aged 2 to <18 years per year after COVID-19, compared to 10.1 before and 14.9 during the pandemic (Table 2).

**Table 2:** Incidence rate of hospitalized children with status asthmaticus between before, during and after COVID-19 pandemic in different age groups.

Age group in years	COVID-19 period		
	Before	During	After
2 to <6	10.1 (6.4–15.2)	14.9 (10.1–21.3)	20.6 (14.6–28.3)
6 to <12	6.2 (3.9–9.3)	3.1 (1.6–5.6)	5.7 (3.5–8.8)
12 to <18	2.1 (0.9–4.4)	1.5 (0.5–3.5)	1.9 (0.8–4.0)
2 to <18	5.7 (4.3–7.5)	5.2 (3.8–6.9)	7.3 (5.6–9.3)

Data are presented as incidence rate per 100,000 children per year (95% CI).

The before COVID-19 period was defined as January 23, 2017, to January 22, 2020 (36 months). The during COVID-19 period was defined as January 23, 2020, to January 22, 2023 (36 months). The after COVID-19 period was defined as January 23, 2023, to January 22, 2024 (12 months).

**Figure 1:**
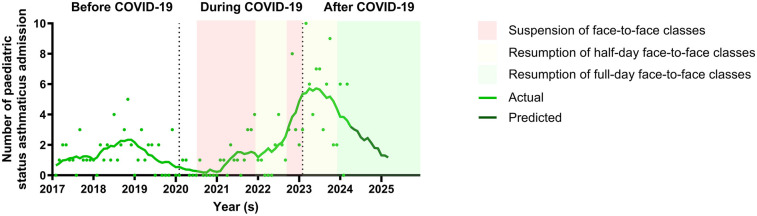
Comparison of monthly hospital admission of children with status asthmaticus from before to after COVID-19 periods. There was a decrease in the monthly hospital admission for children with status asthmaticus at the onset of COVID-19. However, admissions increased following the resumption of half-day face-to-face classes in May 2022 and continued to rise with the return to full-day face-to-face classes in February 2023. The peak in monthly status asthmaticus admission occurred in early 2023, followed by a decline that continued until March 2024. The trend in monthly status asthmaticus admission is predicted to return to before COVID-19 levels by 2025.

Figure 1 showed the correlation between school closures during the pandemic and the monthly hospital admission for children with status asthmaticus. There was a decrease in the monthly hospital admission at the onset of COVID-19. However, admissions increased following the resumption of half-day face-to-face classes in May 2022 and continued to rise with the return to full-day face-to-face classes in February 2023. The peak in monthly status asthmaticus admission occurred in early 2023, followed by a decline that continued until March 2024. The trend in monthly status asthmaticus admission is predicted to return to before COVID-19 levels by 2025. Supplementary Figure 2 further illustrated the changes of paediatric asthma (A) and total pediatric hospitalization (B) admission respectively.

**Figure 2:**
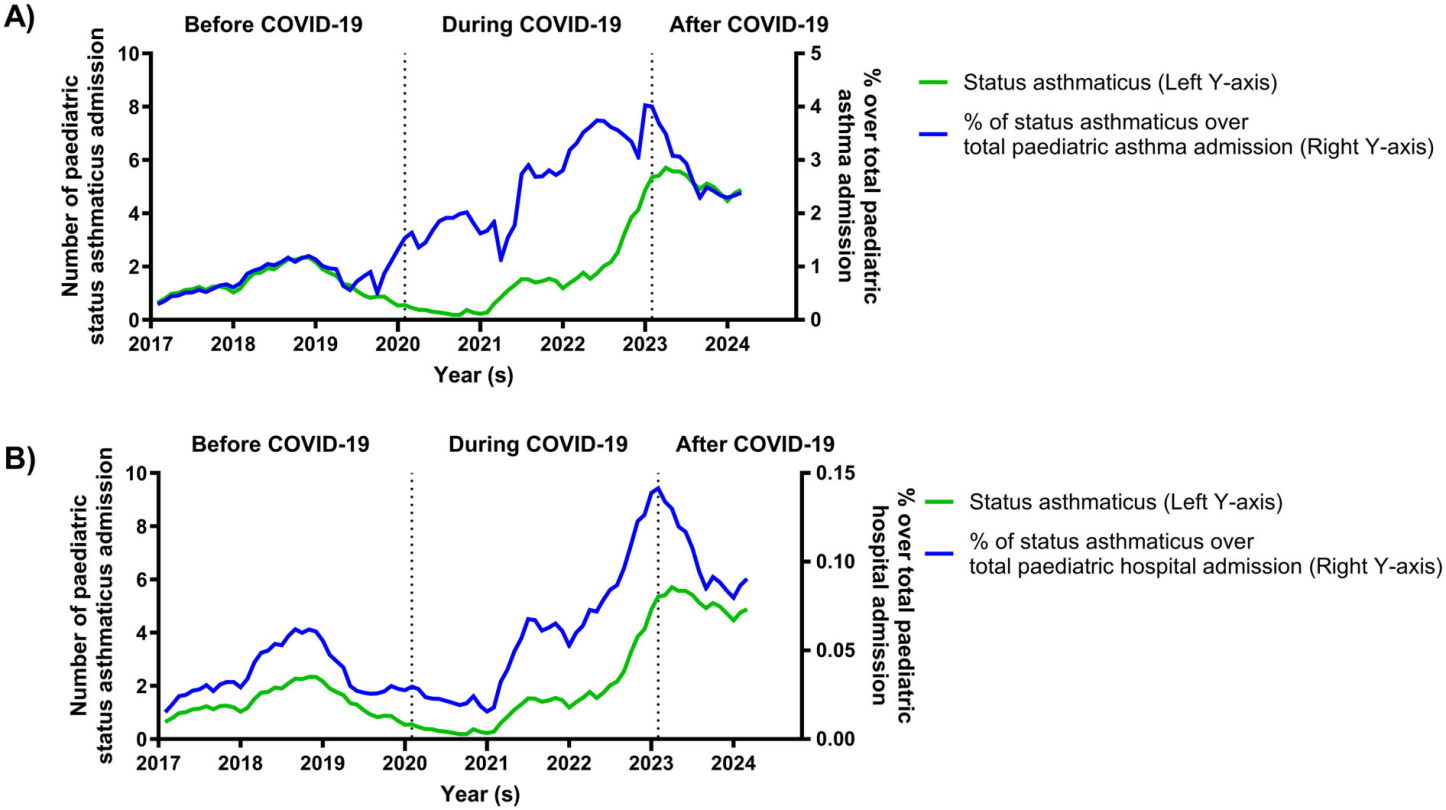
Percentage of paediatric status asthmaticus hospital admission over total asthma and hospital admission from before to after COVID-19 periods. At the onset of COVID-19, the percentage of status asthmaticus admissions over total pediatric asthma admissions increased **(A)**, while the percentage of status asthmaticus admissions relative to total pediatric hospitalizations decreased **(B)**. From early 2021 to early 2023, the percentage of status asthmaticus admissions increased in both categories. However, both percentages declined until March 2024.

After the COVID-19 period, there was a significantly higher percentage of status asthmaticus admissions among total pediatric asthma admissions compared to the period before COVID-19 (2.87% vs. 0.83%, *p* < 0.0001) (Table 1; Figure 2A). Additionally, there was a significant increase in the percentage of status asthmaticus admissions among all pediatric admissions after the COVID-19 period compared to before COVID-19 (0.11% vs. 0.04%, *p* < 0.0001) (Table 1; Figure 2B).

### Secondary outcomes

Additional analysis was conducted on clinical data related to severe asthma exacerbations. There was a higher percentage of oxygen enrichment usage after the COVID-19 period compared to before COVID-19 (84.6% vs. 36.5%, *p* = 0.015) (Table 1). The percentages of other clinical severity parameters in children with status asthmaticus were similar across the three periods, including median length of stay, use of intravenous MgSO4, non-invasive ventilation, invasive ventilation, pneumonia, and death (Table 1).

A subgroup analysis was performed to evaluate if the impact of COVID-19 on status asthmaticus admissions was age-specific (Supplementary Table 1). Children aged 2 to <6 years accounted for the majority of admissions for paediatric status asthmaticus during all three periods (before COVID-19: 44.2%; during COVID-19: 65.2%; after COVID-19: 58.5%) (Supplementary Table 1).

There was an increase in Respiratory Syncytial Virus (RSV) detection after the COVID-19 period compared to during the COVID-19 period (9.2% vs. 0%, *p* = 0.041) (Supplementary Table 2). Additionally, an increase in the co-detection of ≥2 viruses was observed in the post-COVID period compared to the during COVID-19 period (18.5% vs. 4.3%, *p* = 0.040). Rhinovirus (RV) was the most common respiratory virus across all three periods (before COVID-19: 67.3%; during COVID-19: 63.0%; after COVID-19: 69.2%) (Supplementary Table 2). Further analysis showed a comparable percentage of virus detection among patients with status asthmaticus across the different age groups (Supplementary Table 3).

Poisson regression analysis was conducted to examine the risk factors for pediatric status asthmaticus admissions, including environmental factors and COVID-19 periods. After COVID-19 period was identified as an independent risk factor for status asthmaticus admissions [relative risk: 3.43 (95% confidence interval: 1.52–7.73), *p* = 0.003] (Table 3).

**Table 3:** Association of independent risk factors for asthma admission with adjustment by poisson regression.

Risk factors	Relative risk	95% confidence interval	*p*-value
Air quality health index (% of high to serious)	1.17	0.89–1.55	0.266
Humidity (%)	1.05	0.97–1.13	0.225
Temperature (°C)	0.97	0.89–1.06	0.515
Rainfall (mm)	1.00	1.00–1.00	0.508
COVID-19 periods			
Before COVID-19 (2017–2020)	–	–	–
During COVID-19 (2020–2023)	1.26	0.37–4.26	0.708
After COVID-19 (2023–2024)	**3**.**43**	**1.52–7.73**	**0**.**003**

The before COVID-19 period was defined as January 23, 2017, to January 22, 2020 (36 months). The during COVID-19 period was defined as January 23, 2020, to January 22, 2023 (36 months). The after COVID-19 period was defined as January 23, 2023, to January 22, 2024 (12 months).

Bold values indicate *p* < 0.05.

## Discussion

This study evaluated the trend of hospitalizations for pediatric status asthmaticus in PICUs in Hong Kong prior to, during, and after the COVID-19 outbreak. Furthermore, we compared the severity of pediatric status asthmaticus among the three periods and identified the potential risk factors for hospitalization. We also examined the age distribution of the patients and investigated the prevalence of respiratory virus infections in the patients.

Our study revealed an increase in the incidence of pediatric status asthmaticus admissions during the post-COVID-19 period compared to the pre-COVID-19 period. We propose that this rise may be linked to the relaxation of public health measures in Hong Kong following the COVID-19 pandemic, leading to a surge in respiratory virus infections. Notably, a peak in admissions was observed in March 2023, following the resumption of full-day, face-to-face classes in Hong Kong in February 2023. A study on weekly respiratory infection diagnoses at 28 family medicine clinics in Wisconsin showed that for children aged 5–17 years, the risk ratio during the first week of a school session was 1.12 [95% confidence interval (CI) 0.93–1.34], increasing to 1.39 (95% CI 1.15–1.68) in the second week, and reaching 1.43 (95% CI 1.20–1.71) beyond two weeks into a session (17).

Furthermore, we noted a decrease in status asthmaticus admissions at the onset of the COVID-19 period in January 2020, followed by a gradual rise in admissions as half-day, face-to-face classes resumed in early 2021 and 2022. This trend aligns with a study conducted in Hong Kong, which reported a significant reduction in pediatric asthma hospitalizations and respiratory virus isolates during the first year of the COVID-19 pandemic in Hong Kong, which they attributed to the implementation of non-pharmaceutical interventions (10). Non-pharmaceutical interventions extensively adopted in Hong Kong to fight against COVID-19 included the implementation of ordinances on compulsory mask-wearing, border entry restrictions, quarantine and isolation of cases, as well as social distancing measures such as work from home policies, school suspension, and limitations on visiting restaurants and recreational institutions (18). Another observational study involving 2,312 adults with asthma in the UK indicated that the risk of severe asthmatic attacks doubled when social restrictions were eased, implying that the relaxation of COVID-19 measures may correlate with an elevated risk of severe asthma exacerbations (19). We have also analyzed the admission of status asthmaticus related to the SARS-CoV-2 virus, to determine the relationship between the increase in admissions from 2021 to 2023 (during the pandemic) and the severity of COVID-19 disease itself. SARS-CoV-2 virus infection has no significant impact on status asthmaticus admissions. There were 3 cases (6.5%) of status asthmaticus related to the SARS-CoV-2 virus during the COVID-19 periods and 1 case (1.5%) of status asthmaticus related to the SARS-CoV-2 virus after the COVID-19 period.

We observed a greater increase in the incidence of status asthmaticus in preschool children (aged 2 to under 6 years) after the COVID-19 period compared to before COVID-19. We speculate that limited viral exposure during COVID-19 likely prevented young children to acquire immunity against such viruses, making them susceptible to subsequent infection after social distancing measures lifted off (20). Immunity debt implies the lack of protective immunity caused by prolonged low exposure to a specific pathogen. Once preschool children were exposed to respiratory viruses after the COVID-19 periods, an exaggerated response with severe asthma exacerbation resulted. This concurred with a study conducted in Amsterdam on PICU admissions for severe asthmatic exacerbation which reported that during the peak period of COVID-19 in 2021, respiratory viruses were the most frequent trigger for severe asthma exacerbation. When public health restrictions were lifted, children were exposed to respiratory viruses again, which explained the high rate of severe asthma exacerbation during the peak period of COVID-19 in 2021 (3).

An important finding in the current study was the gradual decline in status asthmaticus cases following the peak of admissions in March 2023, although they have not yet returned to pre-COVID-19 levels. Based on the current data, we anticipate that admissions will return to pre-COVID-19 levels by 2025. Our hypothesis is that within 1–2 years, the majority of young children will have acquired immunity against most of the respiratory pathogens from exposures. The immune debt will also be addressed through health measures such as immunization, health education, and pathogen monitoring (21).

Our study revealed a significant increase in RSV detection and the presence of two or more viruses in pediatric patients with status asthmaticus after the COVID-19 period compared to during the pandemic. This might have relation with the surge of status asthmaticus admission after COVID-19 periods. This finding is consistent with a study on viral prevalence patterns from post-COVID era in children in Hong Kong, which showed that in the post-COVID period, the overall virus diversity resumed, and the ratio of co-detection of multiple viruses against a single virus between pre and post COVID-19 periods changed. More cases of co-detection were found in toddlers (1 to <3-year-old) and preschool children (3 to <6-year-old) but not in other age groups (22). Additionally, we identified RV as the most common respiratory virus detected in children with status asthmaticus across all three periods. This finding is in line with previous studies suggesting that RV is frequently linked to asthmatic exacerbations and lower respiratory tract infections. Furthermore, RV is an important contributor to hospitalizations among children in Hong Kong (23). The Childhood Origins of Asthma Study (COAST) also reported that RV was the primary cause of over two-thirds of 217 exacerbations in children (24).

We observed a significant increase in the percentage of status asthmaticus admissions compared to total pediatric asthma admissions, with patients admitted for status asthmaticus post-COVID-19 showing a higher percentage of oxygen utilization. These results indicate a more severe manifestation of asthmatic exacerbation in children following the COVID-19 pandemic. This finding lends support the possible impact of the training of innate immunity, which is acquired by exposure to infections in the first years of life. The lack of immune stimulation due to reduced circulation of microbial agents with non-pharmaceutical interventions imposed by the COVID-19 pandemic likely induced an immunity debt. When exposure to infections occurs after the second years of life, this could result in an intensified response and severe asthmatic exacerbation (20). Severe asthma exacerbations could also be linked to the Hygiene Theory, which proposes that the prevalence of allergic diseases may be associated with infectious diseases, and a lack of early childhood infections could lead to an increased risk of allergic disorders like asthma (25). Previous research by Braun-Fahrländer et al. demonstrated that exposure to microbial products in the first year of life may offer protection against asthma and atopy in children (26). This corresponds with our study's findings of a notable increase in status asthmaticus incidence among preschool children during the COVID-19 pandemic. However, due to the small patient population in our study, a prospective follow-up study focusing on evaluating the immunological aspects of asthma exacerbations post-COVID-19 would be crucial. Another possible explanation for these results could be the shift in healthcare-seeking behavior during the COVID-19 period. Globally, there was a substantial decrease in pediatric healthcare visits during COVID-19 lockdowns, with attendance reductions in Europe ranging from 31% to 85% (27). A study from Hong Kong indicated an overall reduction in all-cause hospitalizations in 2020 compared to 2010–2019. The overall hospitalization reduction (per 100,000 population) for respiratory disease was 632 (95% CI: 607, 658) (28). It is possible that parents opted to bring children with mild asthma exacerbations to private hospitals to avoid contact with COVID-19 patients in public hospitals, potentially leading to a relative increase in the percentage of status asthmaticus admissions compared to total pediatric asthma admissions.

This study also demonstrated a significant increase in the percentage of status asthmaticus admissions among all pediatric admissions, indicating that the rise in monthly pediatric status asthmaticus hospital admissions after COVID-19 could not be attributed to changes in health-seeking behavior after the pandemic.

There was a rise in total pediatric hospital admission after COVID-19 period. A retrospective cohort study measured seasonal patterns of pediatric hospitalizations, pediatric intensive care unit (PICU) admissions diagnosed with acute respiratory diseases during pre-pandemic, COVID-19 pandemic, and late/post-pandemic periods in Canada showed that in winter 2022–2023 (late post-pandemic period), hospitalizations and PICU admissions for bronchiolitis significantly exceeded expected levels based on pre-pandemic trends. Admissions for influenza-like illness, pneumonia, and asthma exacerbation all exceeded expected levels, though not significantly. By winter 2023–2024, hospitalizations and PICU admissions for ARD overall and individually returned to expected pre-pandemic level (29). It is possible that Hong Kong followed this trend, resulting in a surge in all pediatric admissions after COVID-19. However, a limitation of our study is that we did not analyze the diagnoses of total pediatric hospitalizations, so this is just one possible scenario.

Our study revealed that admission for pediatric status asthmaticus was not associated with changes in climate factors such as humidity, temperature, rainfall, and the AQHI. A previous study conducted in nine major cities demonstrated a reduction in PM_2.5_ concentration due to COVID-19 lockdowns (30). While there is some existing evidence supporting a link between PM_2.5_ concentration and asthma exacerbation (31), our study did not find any correlation between PM_2.5_ concentration and admission rates. However, it is important to note that our study had a short follow-up period for pediatric status asthmaticus admissions after COVID-19. A longer-term prospective study would be necessary to determine the effects of COVID-19 and climate changes on the development of childhood asthma.

The strength of this study is that it collected data from the CDARS of the Hospital Authority, capturing pediatric status asthmaticus admission to all the PICUs in Hong Kong. We believe it included nearly all cases of pediatric status asthmaticus in the region, as severe asthmatic attacks would be transferred to PICUs under the Hospital Authority, due to the absence of PICU services in private hospitals.

There are also some limitations in this study. Firstly, as patients' identifiers were removed from the data in the CDARS, we did not have information on the residential addresses of the participants for assessment of exposure to air pollution. Instead, we used data from different health stations in Hong Kong as a proxy for personal exposure to fine PM_2.5_. Secondly, household risk factors for asthmatic attack for individual patients could not be assessed, for example, environmental tobacco smoke, incense burning, and indoor allergens. Drug compliance to controller therapy for patients with chronic asthma could not be evaluated as well due to the anonymous basis. Additionally, our study was an observational analysis that examined the correlation between the implementation of public health measures such as universal masking and social distancing, and the hospitalization rates for status asthmaticus. It was an ecological comparison study that could not establish a direct cause-and-effect relationship between wearing masks and social distancing with the hospitalization rates for status asthmaticus. Lastly, the sample size is small due to the relatively small population in Hong Kong, the change in the hospitalization rates might be affected by other factors occurring in the area.

In conclusion, there has been a rebound in pediatric status asthmaticus admissions in Hong Kong following the COVID-19 pandemic, especially among preschool-aged children. These patients have required increased oxygen usage, with RV being the most commonly detected respiratory virus. This suggests that public health measures implemented during the COVID-19 pandemic may have limited the exposure to respiratory viruses in the population, which may have had a specific impact on the developmental stage of the immune system. Further research would be valuable to examine the duration required for a status asthmaticus hospitalization to return to pre-pandemic levels and strategies to expedite the resolution of immune debt.

## Data Availability

The datasets presented in this article are not readily available due to privacy and ethical restrictions. Requests to access the datasets should be directed to Ka Ka Siu (siukk@hku.hk).
