# Supplementary material for: Population-based study on hospital admissions for pediatric status asthmaticus: from before to after the COVID-19 pandemic
Source: Front Pediatr. 2025 May 9;13:1534770. doi: 10.3389/fped.2025.1534770 (PMC12098421; doi:10.3389/fped.2025.1534770)
Supplement: Supplementary file 1 [file Datasheet1.docx]

**Supplementary Tables:**

Supplementary Table 1: Age distribution of hospitalized children with status asthmaticus between before, during and after COVID-19 pandemic

|  | COVID-19 period  (number of status asthmatics cases) | | | *p*-value | |
| --- | --- | --- | --- | --- | --- |
|  | Before  (n=52) | During  (n=46) | After  (n=65) | Before vs after | During vs after |
| Age group in years | | | | | |
| 2 to <6 | 23 (44.2) | 30 (65.2) | 38 (58.5) | 0.140 | 0.554 |
| 6 to <12 | 22 (42.3) | 11 (23.9) | 20 (30.8) | 0.245 | 0.521 |
| 12 to <18 | 7 (13.5) | 5 (10.9) | 7 (10.8) | 0.777 | 1.000 |

Data are presented as number (percentage of hospitalized children with status asthmaticus in each period).

The before COVID-19 period was defined as January 23, 2017, to January 22, 2020 (36 months). The during COVID-19 period was defined as January 23, 2020, to January 22, 2023 (36 months). The after COVID-19 period was defined as January 23, 2023, to January 22, 2024 (12 months).

|  | COVID-19 period  (number of status asthmatics cases) | | | *p*-value | |
| --- | --- | --- | --- | --- | --- |
|  | Before  (n=52) | During  (n=46) | After  (n=65) | Before vs after | During vs after |
| Status asthmaticus admission with at least one virus identified | 42 (80.8) | 34 (73.9) | 54 (83.1) | 0.811 | 0.342 |
| Types of viruses | | | | | |
| Rhinovirus | 35 (67.3) | 29 (63.0) | 45 (69.2) | 0.844 | 0.543 |
| Parainfluenza | 10 (19.2) | 3 (6.5) | 8 (12.3) | 0.317 | 0.357 |
| Respiratory Syncytial Virus | 4 (7.7) | 0 (0) | 6 (9.2) | 1.000 | **0.041** |
| Adenovirus | 2 (3.8) | 1 (2.2) | 4 (6.2) | 0.691 | 0.401 |
| Influenza | 3 (5.8) | 0 (0) | 3 (4.6) | 1.000 | 0.265 |
| Human metapneumovirus | 2 (3.8) | 0 (0) | 2 (3.1) | 1.000 | 0.510 |
| SARS-CoV-2 | 0 (0) | 3 (6.5) | 1 (1.5) | 1.000 | 0.305 |
| Co-detection of viruses | | | | | |
| ≥2 viruses | 11 (21.2) | 2 (4.3) | 12 (18.5) | 0.816 | **0.040** |
| ≥3 viruses | 3 (5.8) | 0 (0) | 2 (3.1) | 0.654 | 0.510 |

Supplementary Table 2: Virus distribution among hospitalized children with status asthmaticus between before, during and after COVID-19 pandemic

Data are presented as number (percentage of hospitalized children with status asthmaticus in each period).

The before COVID-19 period was defined as January 23, 2017, to January 22, 2020 (36 months). The during COVID-19 period was defined as January 23, 2020, to January 22, 2023 (36 months). The after COVID-19 period was defined as January 23, 2023, to January 22, 2024 (12 months).

|  | COVID-19 period  (number of status asthmatics cases with  at least one virus identified) | | | *p*-value | |
| --- | --- | --- | --- | --- | --- |
|  | Before  (n=11) | During  (n=2) | After  (n=12) | Before vs after | During vs after |
| Age group in years |  |  |  |  |  |
| 2 to <6 | 8 (72.7) | 2 (100) | 10 (83.3) | 0.604 | 1.000 |
| 6 to <12 | 3 (27.3) | 0 (0) | 2 (16.7) | 0.604 | 1.000 |
| 12 to <18 | 0 (0) | 0 (0) | 0 (0) | 1.000 | 1.000 |

Supplementary Table 3: Status asthmaticus admissions with at least one virus identified before, during, and after the COVID-19 pandemic, stratified by age group

Data are presented as number (percentage of hospitalized children with status asthmaticus in each period).

The before COVID-19 period was defined as January 23, 2017, to January 22, 2020 (36 months). The during COVID-19 period was defined as January 23, 2020, to January 22, 2023 (36 months). The after COVID-19 period was defined as January 23, 2023, to January 22, 2024 (12 months).

|  | COVID-19 period  (number of status asthmatics cases with  at least two viruses identified) | | | *p*-value | |
| --- | --- | --- | --- | --- | --- |
|  | Before  (n=42) | During  (n=34) | After  (n=54) | Before vs after | During vs after |
| Age group in years |  |  |  |  |  |
| 2 to <6 | 20 (47.6) | 24 (70.6) | 34 (63.0) | 0.151 | 0.500 |
| 6 to <12 | 17 (40.5) | 7 (20.6) | 15 (27.8) | 0.200 | 0.614 |
| 12 to <18 | 5 (11.9) | 3 (8.8) | 5 (9.3) | 0.744 | 1.000 |

Supplementary Table 4: Status asthmaticus admissions with at least two viruses identified before, during, and after the COVID-19 pandemic, stratified by age group

Data are presented as number (percentage of hospitalized children with status asthmaticus in each period).

The before COVID-19 period was defined as January 23, 2017, to January 22, 2020 (36 months). The during COVID-19 period was defined as January 23, 2020, to January 22, 2023 (36 months). The after COVID-19 period was defined as January 23, 2023, to January 22, 2024 (12 months).


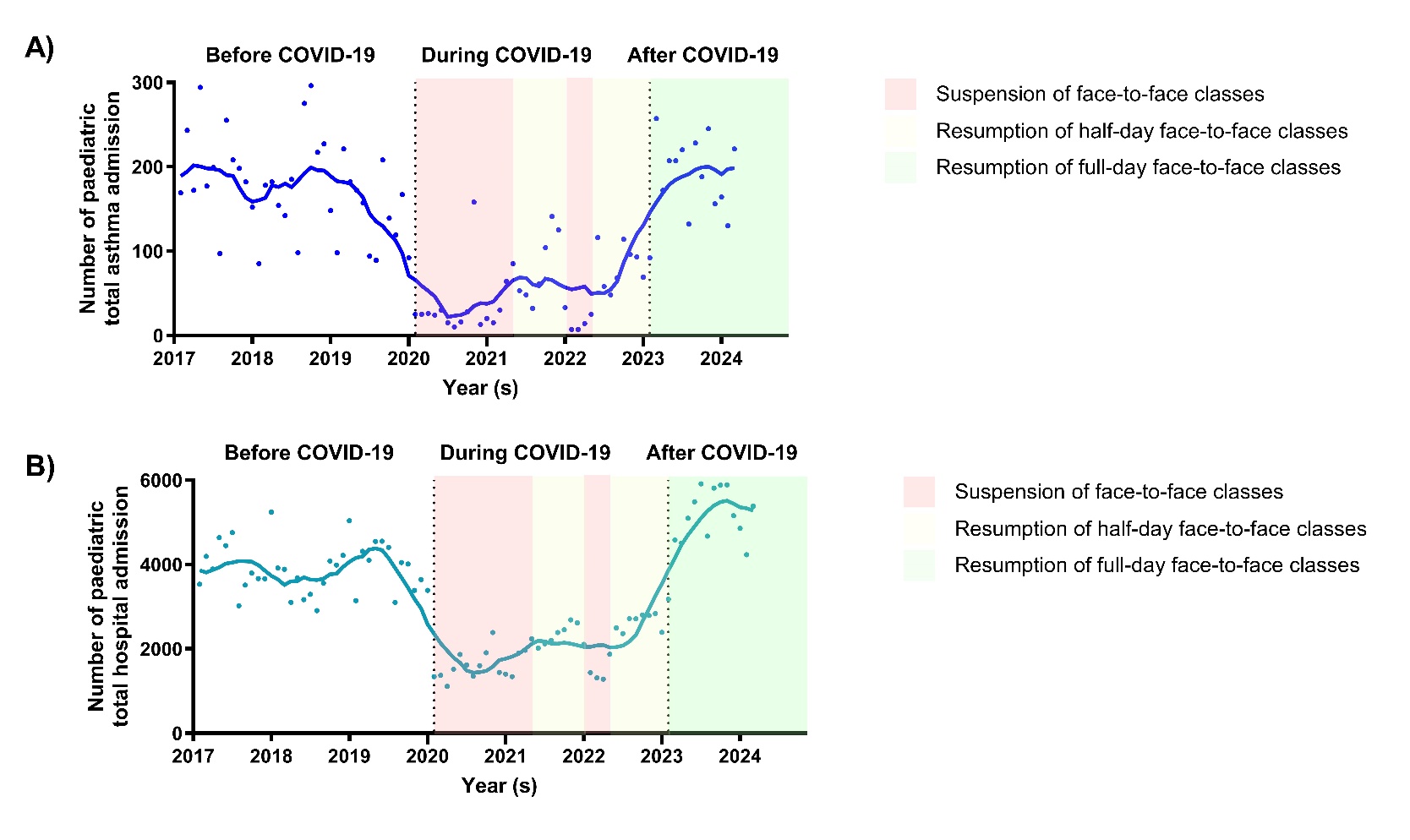
Supplementary Figure 1: Comparison of monthly paediatric asthma and total paediatric hospitalization admission from before to after COVID-19 periods

Both monthly paediatric asthma (A) and total pediatric hospitalization (B) admission followed a similar pattern. There was a decrease in the number of admissions for both paediatric asthma and total hospitalizations at the onset of COVID-19. However, admissions increased following the resumption of half-day face-to-face classes in May 2022 and continued to rise with the resumption of full-day face-to-face classes in February 2023. The peak for both admissions occurred in early 2023, followed by a decline that continued until March 2024.
